# Play it again, but more sadly: Influence of timbre, mode, and musical experience in melody processing

**DOI:** 10.3758/s13421-024-01614-8

**Published:** 2024-08-02

**Authors:** James Armitage, Tuomas Eerola, Andrea R. Halpern

**Affiliations:** 1 Music Department, Durham University, Durham, DH1 3RL UK; 2 Psychology Department, Bucknell University, Lewisburg, PA 17837 USA

**Keywords:** Emotion, Cue, Mode, Timbre, Memory

## Abstract

The emotional properties of music are influenced by a host of factors, such as timbre, mode, harmony, and tempo. In this paper, we consider how two of these factors, mode (major vs. minor) and timbre interact to influence ratings of perceived valence, reaction time, and recognition memory. More specifically, we considered the notion of *congruence*—that is, we used a set of melodies that crossed modes typically perceived as happy and sad (i.e., major and minor) in Western cultures with instruments typically perceived as happy and sad (i.e., marimba and viola). In a reaction-time experiment, participants were asked to classify melodies as happy or sad as quickly as possible. There was a clear congruency effect—that is, when the mode and timbre were congruent (major/marimba or minor/viola), reaction times were shorter than when the mode and timbre were incongruent (major/viola or minor/marimba). In Experiment 2, participants first rated the melodies for valence, before completing a recognition task. Melodies that were initially presented in incongruent conditions in the rating task were subsequently recognized better in the recognition task. The recognition advantage for melodies presented in incongruent conditions is discussed in the context of desirable difficulty.

## Introduction

Music, like all the arts, is a powerful vehicle to communicate emotion. Because it unfolds over time, some aspects of music are similar to verbal arts like theater or poetry, but music also by definition comprises acoustic dimensions that can be perceived at a moment in time, like the visual arts. Those multiple pathways to the emotional message may account for the ubiquity of music across cultures, the appeal of music to people of diverse backgrounds within cultures, and the many situations in which music is voluntarily experienced, especially in the modern age of music-on-demand.

Our focus in this paper is on how two musical cues to valence, the mode (the major or minor scale that the melody is based on) and the instrumental timbre, are integrated (or not) as listeners process melodies for the first time and subsequently remember them. Some prior research has examined how various musical cues convey emotion separately and in combination. One commonly studied cue to emotion is speed, or tempo. For instance, Gagnon and Peretz (2003) found that tempo was the more reliable predictor of valence compared with mode, as rated on a happy/sad scale, and Eerola et al. (2013) also showed tempo, and to a lesser extent, timbre, were used reliably by people to classify a tune into one of four emotions, including happy and sad. (Here we note that our concern here is with the emotion *perceived in*, not *felt from* music. Although cues to felt emotion can also be studied (Chen et al., 2020), these are separable responses to music).

Our current purpose was to contrast two non-temporal cues that differ in interesting ways: mode, as will be explicated more below, is not apparent immediately upon listening to piece, but must be inferred once sufficient notes from the underlying scale are sounded. In contrast, instrumental timbre is evident from the first note, allowing us to compare a more immediately apparent compared with a more dynamic cue. We were also interested in comparing responses among people with more vs. less musical training. Finally, our study is novel in that we measure sensitivity to valence in multiple ways. We collected overt judgments of valence using rating scales, which capture a conscious, deliberative response (Experiment 2). But we also asked for rapid categorical happy/sad judgments, to capture more automatic processing and allow us to assess ease of judgment as reflected in speed of response (Experiment 1). A third way to capture the influence of these cues to valence is to consider the longer term and more indirect measure of recognition memory. Thus the rating task in Experiment 2 also served as an encoding phase of an incidental memory task, as it was followed by a surprise recognition task. Our interest there was the extent to which incongruent versus congruent combinations of cues would affect memory success. For instance, if incongruent combinations such as a minor tune played on a happy instrument improved memory relative to a minor tune played on a sad instrument, that would suggest enhancement of attentional focus when presented with the less expected valence combinations.

We first explain a bit more about mode as a cue to valence. Major and minor modes are frequently associated with happiness and sadness, respectively, in Western music. As mentioned earlier, the mode of a melody derives from the underlying scale, or the building blocks from which the melody notes are selected. Major and minor scales differ with respect to the size of third scale step (minor vs. major third), and the sixth and sometimes seventh scale steps (these differences are all a half-step in magnitude, the smallest possible musical distance—this is the difference between a white key and adjacent black key on a piano). For instance, the major scale is used in *Frère Jacques* (the first three notes of that melody happen to be the first three notes of the scale). Minor mode is less common in many genres of Western music, but some examples are the Beatles tune *Eleanor Rigby* and the folk tune *Greensleeves/What Child is This.* Although the difference in the scales only depends on a few notes, so are small differences in acoustic terms, even nonmusicians in Western musical cultures reliably rate minor tunes as sad and major tunes as happy (Crowder, 1985; Dalla Bella et al., 2001; Leaver & Halpern, 2004).

Instrumental timbre also contributes to the emotional message of music. As Huron et al. (2014) put it in the title of their article (quoting Steve Martin), “You can’t play a sad song on a banjo,” and a number of researchers have verified the association of certain instruments with happiness (the banjo alluded to above) and sadness (for instance, the English horn). A number of acoustic cues that convey emotion in an instrument have been documented, such as attack time (Hailstone et al., 2009), sensory roughness and spectral flux (Eerola et al., 2012; Bowman & Yamauchi, 2016), and loudness and brightness (Eerola et al., 2013; Huron et al., 2014).

Although association of the mode and timbre with emotion has been established, we do not know how these two very different cues may be integrated during listening. More precisely, we were interested to see if the two cues would combine, and if so, additively or multiplicatively. To investigate this, we used a set of tunes devised for prior research (Halpern et al., 2008). All stimuli were derived from actual tunes (some originally in major mode and some originally in minor mode), and had been normed to be unfamiliar, equally musical in each mode, and highly indicative of their respective mode. Each major tune had a minor counterpart, which was identical except for the changed third (or sixth) musical interval. Each was implemented in two timbres that we verified in pretesting as conveying happiness (marimba) and sadness (viola). This therefore created tunes that were congruent for valence cue deriving from mode and timbre, or incongruent, but otherwise matched on many other dimensions. Our first question was whether mode or timbre would be more influential in conveyance of emotion, depending on how we interrogated that response. Pilot work had suggested that when people were allowed to hear the whole tune and then give a valence rating, the cues carried equal weight in conveying emotion. However, in a speeded task, we considered the possibility that the note-constant cue of timbre might be a more powerful influence on responses than mode, given that the timbre is evident from note 1. Thus, timbre could prime the listener to expect a minor mode if for instance a viola starts playing, so that the reaction time to congruent items, which can be determined as soon as the first note that signals mode (the critical note, or CN) is played, might result in even faster RTs for congruent items, compared with the unspeeded rating task.

Considering the variable of musical experience, our interest in this came partly from two prior EEG studies (Centanni et al., 2020; Halpern et al., 2008) that tracked evoked potentials to the CN in these melodies varying in mode. A striking result in both studies was that a late positive component (LPC; at about 550 ms) was observed to the first CN in minor melodies among musicians, which was absent (Halpern et al., 2008) or much diminished (Centanni et al., 2020) among nonmusicians. The LPC is usually considered to be reflective of an attentiveness response, including emotional attentiveness (Liu et al., 2012). As we noted above, as minor mode tunes are less common in Western music, we interpreted this outcome as greater neural sensitivity among the musicians to statistical expectations of mode. The fact that neither group in the cited studies showed an ERP response to the major CN was also consistent with the view that the statistical default was major mode despite the equal representation of both modes in the experiments. Interestingly, this group difference occurred despite the fact that both groups reliably rated major tunes as happy and minor tunes as sad (although musicians did differentiate the two categories more than the nonmusicians). In fact, in the more recent study, a classification algorithm reliably assigned participants to musician or nonmusician groups just based on that LPC amplitude. In the current study, we were therefore interested in whether the relative influence of mode and timbre would be weighted to the former among musically trained individuals across the tasks. As an example, if musicians are more sensitive to mode than nonmusicians, then we might see mode accounting for more variance over timbre in valence ratings. We also entertained the possibility that group differences would be more evident for minor compared with major mode, given the EEG evidence above.

Turning to the other manipulated cue, we have some evidence that even nonmusicians are sensitive to timbre cues to valence. For instance, Goydke et al. (2004) showed that violin versus flute timbre differentially affected the peak latency of the mismatch negativity response in nonmusicians. More specifically, in another study, the attack time and the spectral center of gravity were timbral cues detected automatically by the brain when presented with synthetic timbre sounds (Caclin et al., 2006). However, we do not know if this sensitivity would be enhanced in musicians, who on the one hand, do not necessarily play the instruments in question, but on the other hand, likely need to attend to timbral cues in many playing situations such as blending within and between sections in an orchestra.

Finally, we were interested whether incidental memory for the melodies would be influenced by valence congruity and musical background. As we noted, in Experiment 2 the valence rating task was used both to collect those data per se but also the task served as an encoding phase for an incidental recognition memory task. We were interested in the extent of memory enhancement in the incongruent pairings (minor mode/happy instrument and vice versa) compared with congruent. A fairly robust influence on memory is distinctiveness: all else being equal, we remember distinctive items better than nondistinctive, as per the original Levels of Processing framework (Craik & Lockhart, 1972). This is likely because attentional resources are used to process the incongruity, leading to more robust memory representations. For instance, Guillaume et al. (2020) presented participants with congruent object/scene (a tent in a field) or incongruent (a shower cabin in a field), while EEG was being recorded. When later presented with just the object, participants were better able to recall the associated scene better if it had been in an incongruous compared with congruous setting. This pattern was associated with a large N400 response to the incongruous scenes during encoding (the N400 is consistently associated with a response to unexpectedness). We also know that similar EEG responses are observed during music listening when incongruity is introduced in the form of an unexpected note or harmony (Carrus, et al., 2013). A recent direct test of memory for congruence effect involving music and facial expressions showed that emotionally incongruent pairing (happy music with sad facial expression) led to higher retrieval confidence than emotionally congruent stimuli (Panteleeva et al., 2021).

Although Carrus et al. (2013) did not test for an advantage for incongruous items in old/new recognition, other studies have done so. For instance Michelon et al. (2003) showed participants objects that were mashups of two objects, such the head of a key on the left side of the object, fused with an image of a snake instead of the thin part of the key. Incongruous pictures were better recognized, and fMRI results showed increased activity in many visual and parietal areas during encoding of incongruous versus congruous items. In some memory research contexts, the better recall of incongruent objects is explained via the notion of *desirable difficulty* (Bjork, 1994; Yue et al., 2013), which relates to fluency of processing. Less fluently processed items such as slightly blurred text is presumed to be better remembered due to emphasis given to the initial learning process of this challenging information. Although the jury is still out on the explanatory robustness of desirable difficulty in memory (see Geller et al., 2018), and very few studies have looked at auditory objects, the theory offers one possible mechanism to explain the perhaps-counterintuitive results concerning better recognition of incongruent items.

In the musical domain, Schwartz et al. (2020) considered recognition memory for melodies in pianists. Recognition memory was assessed following a sight-reading paradigm in which melodies were played in a congruent (i.e., treble clef–right hand or bass–clef left hand) condition or incongruent (treble clef–left hand or bass clef–right hand) condition. They demonstrated that memory for melodies was better in the incongruent condition, attributing the advantage to desirable difficulty. Many of the extant studies on incongruity operationalize that variable as a very rare event, or a bizarre or even incorrect combination. For instance, in past research, an incongruent item in melody might be one in which participants hear a wrong harmony or mistuned note (Brattico et al., 2009). The type of incongruity in our study is more subtle: one could very well hear a sad melody played on a marimba, perhaps as an artistic choice of the composer, but it is somewhat unexpected. We wanted to see if this type of affective mismatch would enhance recognition memory, presumably by calling attention to the mismatch as a tune began in one timbre, but then sounded the note signaling the mismatched mode. Furthermore, we were interested in whether the predicted incongruency advantage would be enhanced among musicians, given their documented greater sensitivity to mode differences, and our speculation about more sensitivity to timbral differences as well.

## Experiment 1

The aim was to examine how combining two emotional cues, mode and timbre, contribute to recognition of emotional expression using a response time task. We also wanted to explore whether this recognition is influenced by musical experience as it has been recently demonstrated that musicians have a more differentiated neural and behavioral response to mode (Centanni et al., 2020) and timbre (Caclin et al., 2006) than do nonmusicians.

### Methods

#### Participants

We estimated the sample size using past studies using response times (RTs) for happy/sad decision across mode and musical expertise. These studies showed Cohen’s *d* from 0.53 (an overall difference in RTs between musicians and nonmusicians) to 0.62 (difference between major and minor for musicians in Halpern et al., 2008) and to 0.39 and 0.32 (difference within musicians and nonmusicians, respectively, across the mode in Centanni et al., 2020). To obtain a power of 0.80 with significance level of 0.05, with an average effect size (*d* = 0.465) from these previous studies, we needed 38 participants in both groups (musician/nonmusician). Participants were recruited from Prolific.co (see Palan & Schitter, 2018) using criteria that specified native English speakers who currently live in the UK to keep the language, education, and terminology homogenous. As the musical expertise cannot be selected for in these participants, we ended up overrecruiting to achieve the required number of musicians, resulting in 92 participants (53 females), where 39 were musicians and 53 nonmusicians. We defined nonmusicians and musicians based on a combination of several criteria (musicians having more than 5 years of instrument training, more than 1 hour of practice per day, 1+ instruments played, more than 3 years in music theory training, and also a self-nomination into musician/nonmusician). The mean age of the sample was 33.80 years (*SD* = 13.28) and education was largely consistent with the UK population : 4% had GCSEs or equivalent (high school to age 16), 32.6% had A-Levels or equivalent (high school to age 18), 39.1% had attained an undergraduate degree, 22.8% had a postgraduate degree, and 1.1 % held a doctoral degree.

#### Stimuli

Fourteen unfamiliar melodies both in major and minor mode were chosen from previous research (Halpern & Martin, 2008). Half of these were in major, half in minor mode, and were constructed in pairs so that they were otherwise identical (equally musical etc. see Halpern et al., 2008). In each melody, an early note (third or sixth degree of the scale) identified the mode as being major or minor. These critical notes (CN) typically occurred between the first 500 ms and 1,700 ms in these melodies (25th percentile of 539 ms, 50% of 1,003 ms, and 75% of 1,653 ms).

Two timbres were chosen to represent happy and sad expression. Seven candidate timbres to represent high capacity to express sadness (viola and Vienna horn), happiness (cembalo and marimba), and neutral (piano, and two synthesizer sounds) were identified based on past studies (Eerola et al., 2012; Huron et al., 2014). To assess these candidates, a simple ascending and descending triadic sequence lasting 5 s was created and rendered with all instruments. Vienna Symphonic Library was used with viola and Vienna horn, utilizing a sustained articulation, cembalo, marimba, and piano (Bosendörfer) instruments utilizing their standard articulation, and the two synthesiser sounds, Dream Dancer and Vintage Doppler were from Logic Pro X library. All sounds were equalised to subjective levels of loudness (peak Sone level) and 10-ms fade-ins and outs were inserted to avoid clipping. The sound clips were presented in random order to participants who rated them in terms of emotional valence ranging from happy to sad, familiarity and pleasantness using 7-point Likert scale. Twenty participants (nine women, mean age 25.0 years, *SD* = 3.6) who were living in the UK and were native English speakers were recruited from Prolific.ac (online recruitment service). The musical expertise of the participants represented the population at large, most had not studied any music (number of years of private music tuition was 1.15, *SD* = 2.3), and the majority classified themselves as music-loving nonmusicians (56%) or nonmusicians (20%), but there were some self-classified amateur musicians (20%) and one semiprofessional musician. The results showed that marimba was the happiest instrument (*M* = 4.75, CI [3.92, 5.08]) whereas the viola (*M* = 2.65, CI [2.07, 3.23]) conveyed the most sadness and these two clearly differed in terms of the ratings, *t*(114) = 4.00, *p* < .001. Piano, viola and marimba received the highest familiarity ratings and were also equally pleasant (three separate contrasts, all n.s.), so they were selected as the sounds for the actual experiment. These two sounds also maximize the differences in timbre dimensions (attack time was 16ms for marimba and 260 ms for viola) and spectral centroid: only 3% of the spectral energy was above 1500 Hz for marimba compared with the same pitch in viola, which had 24% of the spectral energy above 1500 Hz. These differences should be perceptually relevant for listeners even when the sounds are not the focus of attention (Caclin et al., 2006).

All melodies were rendered with marimba and viola and to create congruous (major mode and marimba or minor mode and viola) and incongruous (major mode, viola timbre, or minor mode and marimba) combinations of the melodies, yielding (14 × 4) 56 stimuli altogether. The exported audio files were normalized to the same perceptual loudness level using the peak sone level. The stimuli, experiment interface, and data are available here: https://osf.io/qym83/?view_only=ed20c82899264b9aad70b0fa5fbe2cd3

#### Procedure

A timed judgment task was utilized where participants heard the melody and their task was to indicate as quickly as possible whether the music was happier or sadder with a key press (“H” or “S” on their keyboard), where “H” referred to happier and “S” to sadder. We chose the relative terms to acknowledge that the incongruent conditions would likely convey emotions that were not unequivocably happy or sad (see also De Jong et al., 2000). They were given five practice trials, and then 56 actual experiment trials in random order. Participants answered questions about their musical expertise, education, age and gender. Two musical expertise questions (musical training Questions 3 and 4) were derived from Goldsmiths Musical Sophistication Index (Müllensiefen et al., 2014), and the rest of the questions asked about how many years they had engaged in instrument training, how many hours per day this practice took at its peak, the number of years of theory training, and how many instruments the participant played. The data were collected online using PsyToolkit (Stoet, 2017) that provides accurate RT data, even in an online setting (Armitage & Eerola, 2020). The experiment took approximately 13 minutes to complete, and the participants were reimbursed for their time (£8.50/h). An institutional ethics approval was obtained.

### Results

**Fig. 1:**
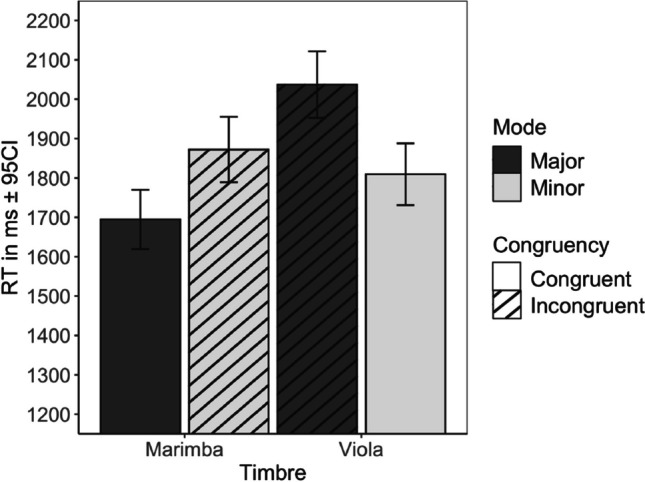
Means and 95% confidence intervals of the response times relative to CN across mode and timbre

**Fig. 2:**
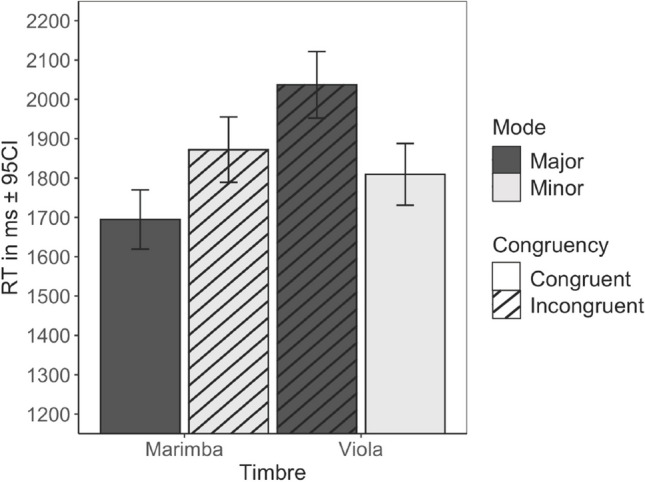
Means of valence across musical experience and mode and timbre

We eliminated incorrect responses to congruous stimuli (5.7%) and responses made 5 s after and 2 s prior to the critical note in the melody, eliminating a further 7.6% of observations (both in line with reaction time analysis recommendations; see Ratcliff, 1995; Spruyt et al., 2012). Of the remaining responses, 47.9% of the valid happier/sadder decisions were in the fully congruent stimulus combinations and 52.1% were in incongruent combinations. In the incongruent conditions, 55.1% of happier/sadder decisions (28.9% of all decisions) were consistent with mode and 44.9% (23.4% of all decisions) were consistent with timbre. The RTs relative to the CN were analyzed with linear mixed models, with instrument/mode congruence and musical experience as fixed factors and participant as a random factor. This yielded a significant main effect of congruency (*β* = 245.0), *t*(4375) = 5.22, *p* < .001, but no significant effect of musical experience (*β* = 136.7), *t*(98) = 0.81, *p* = .42, nor an interaction between musical experience and congruence (*β* = 34.6), *t*(4376) = 0.49, *p* = .63. Melodies with congruent cues were processed more rapidly (*M* = 1,770 ms, *SE* = 83 ms) than those with incongruous cues (*M* = 2030 ms, *SE* = 83 ms). To explore the determinants of the congruence in more detail, we ran the analysis with mode and timbre and musical experience as fixed factors, and participants as a random factor. This analysis produced significant main effects of timbre, *t*(4371) = 6.03, *p* < .001, and mode, *t*(4371) = 3.43, *p* < .001, but not musical experience, *t*(116) = 1.08, *p* = .283. Moreover, there was an interaction between timbre and mode, *t*(4371) = 5.20, *p* < .001. Figure 1 illustrates the RT pattern for all conditions. Regarding the congruent conditions, although faster responses were given to marimba melodies in major mode (*M* = 1,695 ms, *SE* = 38 ms) and slower responses to viola melodies in minor mode (*M* = 1,809 ms, *SE* = 40 ms), this difference was not significant, *t*(4375) = 1.67, *p* = .333, with correction for multiple testing with Tukey’s method. Turning to incongruent combinations, the slower mean response time was obtained with viola in major mode (*M*=2037 ms, *SE*=43 ms; the slowest of all four conditions), which was significantly slower than the other incongruent pairing, marimba in minor mode (*M* = 1,872 ms, *SE* = 42 ms, *t* = −3.37, *p* = .0043) Fig. 2.

### Discussion

The predicted effect of congruence was clearly evident as melodies with congruent emotion cues were processed 260 ms faster than melodies with incongruent cues. Timbre produced larger effects on response times than mode but both cues contributed to the response time difference. The results are largely consistent with past studies that have established that mode and timbre contribute to the expression of emotional valence in music (Gagnon & Peretz, 2003; Hailstone et al., 2009). As timbre is present from the onset of the first note of the melody, that could account for its greater influence, as in that sense it functions as a valence prime. The results are also partially consistent with studies exploring the automatic processing of these cues (e.g., Caclin et al., 2006; Centanni et al., 2020; Halpern et al., 2008), although those studies also showed that musical experience moderated and generally amplified the ERP patterns in their data, unlike the current results. It is possible that either the task or the outcome measure (response time) is not sufficiently precise to detect group differences that are seen in the more time-sensitive ERP data (Centanni et al., 2020). Also, if timbre cues are very informative no matter one’s musical background, the greater salience of that cue in our timed response may have overridden potential greater sensitivity of the musicians to the mode cue that has in the past been detected in stimuli where only mode was varied.

## Experiment 2

As Experiment 1 demonstrated differences across mode and timbre in response times to binary classification, we wanted to extend our findings by using two other measures—namely, a subjective valence rating task and an incidental memory task. The purpose of the valence rating task was to clarify how listeners perceived emotional expressions of the stimuli when speed was not emphasized, in particular focusing on the incongruous stimuli that may yield conflicting emotional associations. Considering dominance of timbre over mode in the speeded task in Experiment 1, Experiment 2 served as a partial replication and extension of that finding. Inclusion of a memory component allowed us to see the impact of emotions on another task, and in particular to revisit any group differences (which did not appear in Experiment 1). Perhaps surprisingly, musicians do not show superiority to nonmusicians in standard recognition memory tasks with unfamiliar tunes (see Halpern & Bartlett, 2010, for a review). But the current paradigm might reveal a possible musician memory advantage if they were making more nuanced ratings of the melodies. Secondly, as semantic incongruency is known to increase recognition rates in other memory tasks for reasons discussed earlier, we were interested if this would extend to congruency with respect to the novel domain of musical cues to emotion. For this reason, Experiment 2, had a rating task which was also the encoding phase of the memory task, and the retrieval task, where participants heard a selection of the melodies from the rating task together with new but musically matched melodies to test explicitly assess the participant’s ability to recognize the memories presented in the first phase.

### Methods

#### Participants

Sixty-six participants, recruited via Prolific.co, completed the experiments. Participants received an average of £8.03/h for participating in the study. Data from 11 participants were deleted prior to analysis owing to a high proportion of no responses in either the rating task or memory task. Thus, the overall sample size was 55 participants (30 female, 25 male; mean age = 35.9 years, *SD* = 12.2) The participants’ education levels (8.9% GCSE, 33.9% A-Levels, 39.3% undergraduate degree, 17.9% postgraduate degree) were consistent broadly speaking with those in Experiment 1. Using the same set of criteria as Experiment 1, 25 participants identified as musicians and 30 did not identify as musicians. The sample size is in keeping with recent similar studies (for instance Gryder, 2023; Rainsford et al., 2019; Weiss et al., 2021)

#### Materials

The stimuli comprised melodies taken from the same previously validated collection used in Experiment 1. Forty melodies were used: ten melodies to represent each condition mode by instrument combination, where the happy and sad instruments were marimba and viola, respectively, and the happy and sad modes were major and minor. Musical sophistication was measured using both an in-house questionnaire and the Goldsmiths Musical Sophistication Index (Gold-MSI; Müllensiefen et al., 2014). The experiment was presented in the web-based version of PsyToolkit (Stoet, 2017).

#### Procedure

Prior to the main experiment, participants provided basic demographic information and completed the musical sophistication/experience questionnaire. Participants were informed that they would hear a series of melodies which they would rate for their emotional content and afterward would be asked some questions about the melodies they had just heard. For the rating task, participants heard the 40 melodies presented in a random order and were asked to rate the melodies on a 7-point scale, where 1 represented *sadder* and 7 represented *happier*. As in Experiment 1, the purpose of these relative terms was to facilitate participants’ responses to incongruent items that may otherwise have been challenging to rate as sad or happy. However, the usual Likert scale options were provided as we were not collecting immediate responses. Participants were able to rate each melody at any time during its presentation, but they were not able to move onto the next item until they had heard the complete melody.

After completing the rating task, participants were directed to the memory task. There were four versions of the task, each identical except for using a different subset of the rated stimuli. Different subsets with different counterbalancing were used to ensure that any differences in recognition were down to mode and timbre and not the result of any one melody being more memorable than another. For the memory task, we presented the melodies with a previously unused, emotionally neutral timbre in order to focus on structural identity of melody rather than on timbre. We chose piano as the instrument for the memory task, as piano is a common instrument, fairly neutral in rated valence, and acoustically a hybrid of the two timbral cues maximized in the valenced instruments.

In the memory task, participants heard 32 melodies presented in a piano timbre—a subset of 16 of the 40 melodies that they heard in the rating task (four each from the major-marimba, major-viola, minor-marimba, and minor-viola conditions, labeled hereafter as *targets*), and 16 new melodies (eight major and eight minor melodies that were similar in length and character to the original melodies from the rating task, as *foils*). The melodies were presented in a random order. Participants were asked to rate the melodies on a scale of 1–6, where 1 represented *definitely new* (i.e., unfamiliar) and 6 represented *definitely old* (i.e., familiar). In this recognition task, the main manipulation of interest was *congruency* as defined in Experiment 1.

### Results

#### Valence ratings

As the valence ratings violated the assumption of normality, we carried out an ordinal regression, fitting a cumulative link model (CLM; fitted using the R library ordinal; Christensen, 2023) with two fixed within-subjects factors of instrument and mode, the fixed between-subjects factor musical experience and participants as a random factor; we used a probit link function.

Any main effects and interactions were probed further using planned contrasts with Bonferroni adjustment for multiple comparisons. There was a significant main effect of mode. In line with expectation, major melodies (mean sad/happy rating = 4.53, *SD* = 1.43) were rated as more positive than minor melodies (mean happy/sad rating = 3.11, *SD* = 1.39), *β =* 0.88, *z =* 10.19,* p* < .001. There was also a significant main effect of instrument: planned contrasts revealed that, as predicted, marimba (mean sad/happy rating = 4.57, *SD* = 1.35) was rated as significantly happier than melodies presented in viola (mean sad/happy rating = 3.12, *SD* = .49), *β = 1.22, z =* 14.00, *p* < .001. The main effect of musical experience proved nonsignificant, *p* = .43. Furthermore, the model yielded a significant interaction of mode and musical experience, *β =* 0.65, *z* = 5.19, *p* < .001. Planned contrasts indicated that both musicians and nonmusicians rated minor melodies as sadder than major melodies in line with the main effect. However, there was no group difference between musicians and nonmusicians in happy/sad ratings for major melodies, *t* = 1.859, *p* = .34, and only a marginal difference between groups for minor melodies, *t =* 2.5, *p* = .08. Interestingly, we did see that the main effect was amplified in the musician group compared with the nonmusician group (i.e., the difference in happy/sad ratings between major and minor melodies was greater in the case of musicians than nonmusicians, *t* = 32.7, *p* < .0001). We also saw a marginally significant two-way interaction of instrument and musical experience, *β* = 0.36, *z* = 5.61, *p* < .09: planned contrasts revealed that, whereas marimba was rated as significantly happier than viola by both musicians and nonmusicians, the difference in happy/sad ratings between viola and marimba was greater in the case of nonmusicians than musicians, *t* = 4.450, *p* < .0001. The final interaction of interest was the significant interaction of instrument and mode, χ^2^(1) = 16.7, *p <* .001. Planned contrasts indicated that the difference between the valence ratings for major and minor melodies was significantly greater in the case of viola than for marimba, *t* = 4.2, *p* < .001. The three-way interaction of mode, instrument, and musical experience was nonsignificant, *p* = .212.

#### Memory ratings

Initially, we dichotomized the memory ratings—ratings of 1, 2, and 3 were defined as *new*, whereas ratings of 4, 5, and 6 were defined as *old*. Table 1 summarizes the old and new categorizations by stimulus type: congruent, incongruent, and foils. The difference in criterion scores, *C*, proved nonsignificant, *t*(54) = 0.68, *p* =.50.

**Table 1:** Hit, miss, false-alarm rates, and criterion scores for Experiment 2

	Congruent	Incongruent	Foil
New	45.1 (Miss)	40.7 (Miss)	64.1 (Correct rejection)
Old	54.9 (Hit)	59.3 (Hit)	35.9 (False alarm)
Criterion	0.19	0.13	–

The results of the memory task using the entire rating scale were then analyzed using receiver operating characteristic (ROC) methods (see, e.g., Ratcliff et al., 1992; Swets, 1973; Yonelinas & Parks, 2007). We calculated the area under the curve (AUC) for each participant in both the congruent and incongruent conditions; for this metric, .5 represents chance performance.

**Fig. 3:**
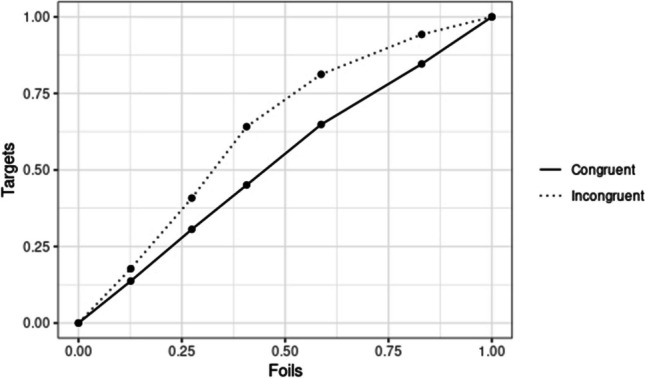
Mean ROC curve for recognition memory task

**Fig. 4:**
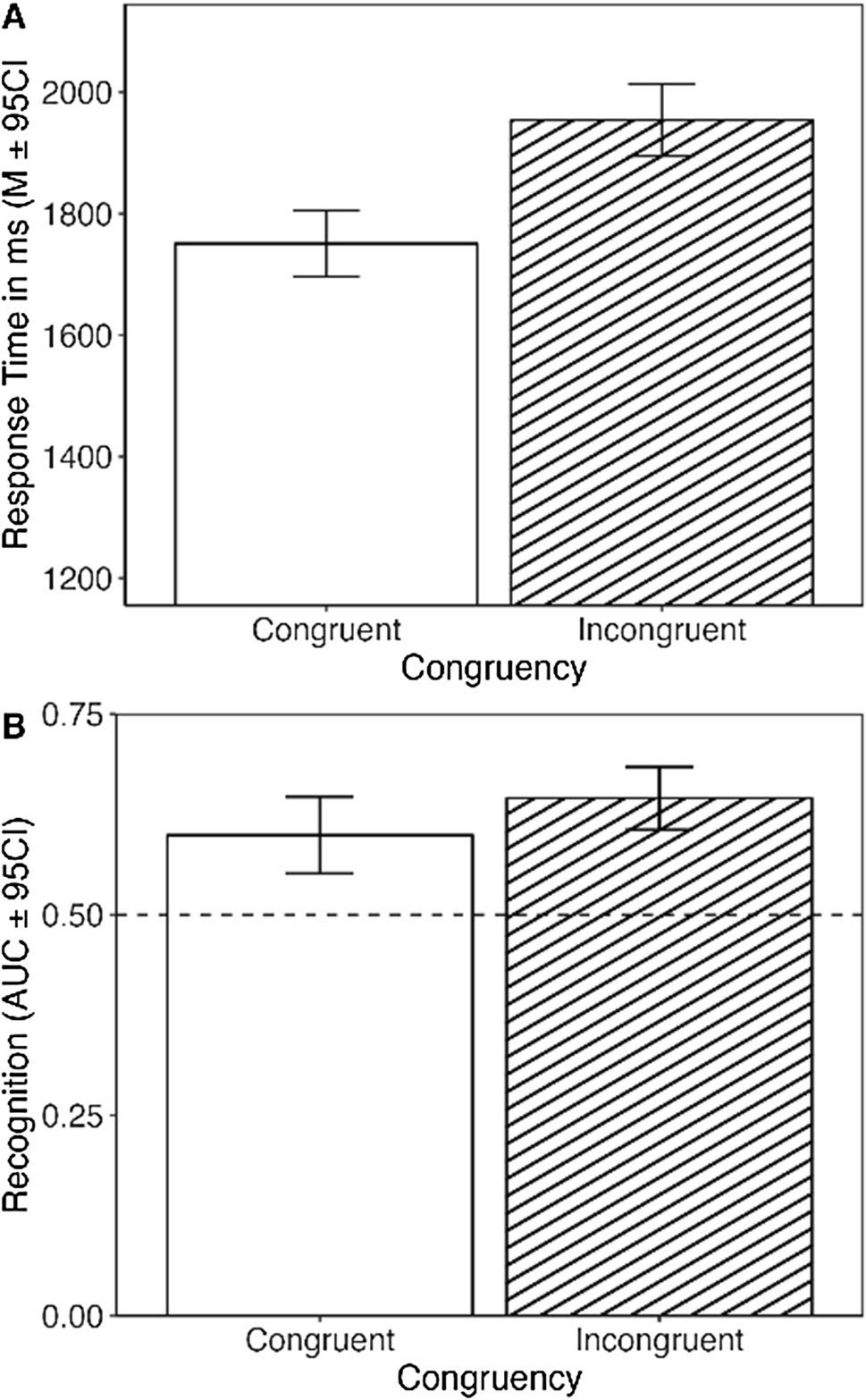
Mean RT and mean AUC by congruency condition

Shapiro–Wilk tests indicated that AUCs were distributed normally, and so we proceeded with parametric tests. A within participants *t* test revealed that AUC values for melodies presented in incongruent conditions (mean AUC value = 0.64, *SD* = 0.15) were significantly greater than those for melodies presented in congruent conditions (mean AUC value = 0.59, *SD* = 0.18), *t*(54) = 2.56, *p* = .013, 95% CI ΔAUC [0.01, 0.08], *d* = 0.52. Figure 3 gives the ROC curves for stimuli presented in incongruent versus congruent conditions. The mean valence judgment reaction times from Experiment 1 are presented in Fig. 4 alongside the mean areas under the curves from Experiment 2.

## General discussion

The valence ratings in Experiment 2 served as a validation and confirmation of the stimuli and replicated results from past studies where the saddest affective connotations are achieved with minor mode and viola timbre and the happiest expression is conveyed with major mode and marimba timbre (Huron et al., 2014). However, cue congruency has rarely been studied with indirect measures such as reaction times and memory performance.

Experiment 1 demonstrated that incongruent cue combinations are processed more slowly than congruent, and musical experience had no impact on the magnitude of this pattern. Timbre produced a larger effect in the response times than mode but mode and timbre operated in additive fashion in affecting times, rather than interactively. This pattern of responses is largely consistent with other studies using self-report of emotion using the same expressive cues (e.g., Dalla Bella et al., 2001; Eerola et al., 2013).

One contrast is with past studies that have found mode to be highly significant cue for musicians but not as much for nonmusicians (Centanni et al., 2020; Leaver & Halpern, 2004). The current data did not demonstrate differences in musical experience groups in the indirect measures (response time or memory tasks) but musicians were more sensitive to mode in the direct self-report task (valence ratings). In contrast to the impediment of incongruency in categorization speed, Experiment 2 showed a faciliatory effect of congruence in an unexpected recognition memory task, with a significantly superior performance with incongruous melodies over congruous melodies.

Taken together, the contrasting effects of congruency on speed and memory suggest that incongruity slows classification time down, perhaps due to the need to inhibit information from one of the two cues given the need for a binary classification. But that additional processing due to incongruence may also make the stimuli more memorable, possibly through increased attentional focus during the encoding stage, as per the desirable difficulty theory (Yue et al., 2013). Indeed, this account agrees with authors such as Krebs et al. (2015) who contend that mild incongruency induces increased top-down attention that inhibits reaction time, but that is associated with better incidental encoding of information about the target stimuli. An alternative explanation is simply that perceptual distinctiveness (see Stangor & McMillan, 1992) makes the stimuli more memorable. An online measure of attention could help support one of these explanations; of course, incongruency could elicit both of these processing scenarios.

Almost all research on musical expression has been done with self-reports, which are valid measures but require fully conscious processing and thus unable to tap into semiautomatic responses in the way the indirect measures can. Here we argue that the indirect measures such as response time and recognition allow to measure the impact of the cues to the processing of this information more directly, or at least in very early processing, compared with conscious self-report method. These indirect measures not only allow insight into the less verbally mediated responses per se, but can be used in studies where reliance on conscious self-report methods might not be ideal for instance, when comparing people across different cultures, or for investigating responses in people with less verbal facility, such as children or persons with dementia. Of course, these objective measures of processing such as ERPs require clear rationale and testable hypothesis to create suitable empirical paradigms. The two indirect measures applied here can generate several testable predictions suited for such an experiment.

We of course acknowledge that the cues chosen in this study were specific to Western musical culture, appropriate for our Western listeners. Mode is a cultural construction (Parncutt, 2013) and is either organized differently in other cultures or can carry entirely opposite affective connotations in some cultures (e.g., minor mode is positively valenced and major is negative in the music of Khow and Kalash tribes in Northwestern Pakistan; Lahdelma et al., 2021). Similarly, various timbres may be associated with happy and sad expressions in Western music, but less so in other cultures. However, timbre might yield more similar responses across cultures than mode because timbral cues to emotion are likely linked to expressive speech and have shown some generalizability across cultures in emotional expression in music (Laukka et al., 2013) and in vocal expression (Laukka & Elfenbein, 2021). In other words, specific cue–emotion associations have been theorized to arise from specific modifiers that emotions have on physiological states, which in turn are audible in speech expression (Laukka et al., 2016; Scherer, 1986). The present study offers an approach that may help future work to capture the impact of the expressive cues and their combinations indirectly, without demand characteristics.

A second logical continuation of the present study would be to compare other cue combinations such as tempo, articulation, or dynamics known to communicate expression in music (Gabrielsson, & Lindström, 2010). We chose mode and timbre for reasons detailed above, but these other cues, arising from very different acoustic manipulations, could allow us to see the generalizability of these indirect responses (Gabrielsson & Juslin, 1996; Hevner, 1935; Laukka et al., 2013).

Processing dysfluency has not previously been explored as a causal mechanism in memory for music. Here, we found better recognition of incongruent melodies, which is consistent with the processing disfluency theory. This link could be explored further by varying the degree of ambiguity created by the contrasting cues more systematically, creating a range of incongruity. Other affective cues could be utilized such as the complexity of melody (Eerola et al., 2016) or other aspects of musical ambiguity (Pressnitzer et al., 2011). Music educators might find these results useful insofar as predicting that pieces their students struggle with a bit more during study might actually promote less score dependency once the piece is learned.

The role of musical experience and training in processing the affective cues to emotions is an interesting question for further study. Here we observed differences in valence ratings of melodies between musicians and nonmusicians but the groups did not differ in the two indirect tasks. While musical experience differences are sometimes found in self-report measures relating to mode (Castro & Lima, 2014), differences are not evident in all studies (e.g., Bigand et al., 2005; Gagnon & Peretz, 2003). Studies utilizing automatic processing indices such as in ERP components have, however, produced clear experience differences in processing of mode between musicians and non-musicians in some indices such as the late positive component, particularly to minor mode (Centanni et al., 2020), or decreased gamma and theta band activity in the right posterior regions for musicians (Jenni et al., 2017). These components suggest that increased experience may be related to selective attention to pitch distribution as a marker of musical syntax rather than suggesting that this is inherently related to affective processing. Changes in timbre, which has generally received less attention than mode, is also able to modulate LPC responses to timbrally incongruent versions of the melody (Zhang et al., 2019).

## Limitations

We end with some limitations to consider for future work. Here we used single line melodies rather than richer, multi-instrument melodies that Western people would likely listen to in real situations. And although they were carefully selected, we did use only two instruments, so expanding to other timbres that are usually considered happy and sad in Western culture would be useful. We collected data online, which may improve ecological validity, but sacrifices lab-style control of the environment. However, numerous memory studies have been carried out in online settings (e.g., Leding, 2019; Lukasik et al., 2019). Also, response times to audio examples can be collected with high temporal precision (Eerola et al., 2021). However, we also note that the stimuli did have some aspects of “real” music. For one thing, the timbres were sampled from actual instruments rather than digitized. And although the melodies were only single lines, most were derived from folk and other extant tunes; some others were composed but designed to mimic that folk tune tradition and all had been rated as being very musical in prior work (Halpern et al., 2008) and thus not only had origins in human composition but had remained in the literature.

## Data Availability

All data and stimuli are available at: https://osf.io/qym83/?view_only=ed20c82899264b9aad70b0fa5fbe2cd3

## Code availability

All analysis scripts and PsyToolkit scripts are available at: https://osf.io/qym83/?view_only=ed20c82899264b9aad70b0fa5fbe2cd3
